# Novel insights into *PORCN* mutations, associated phenotypes and pathophysiological aspects

**DOI:** 10.1186/s13023-021-02068-w

**Published:** 2022-01-31

**Authors:** Annabelle Arlt, Nicolai Kohlschmidt, Andreas Hentschel, Enrika Bartels, Claudia Groß, Ana Töpf, Pınar Edem, Nora Szabo, Albert Sickmann, Nancy Meyer, Ulrike Schara-Schmidt, Jarred Lau, Hanns Lochmüller, Rita Horvath, Yavuz Oktay, Andreas Roos, Semra Hiz

**Affiliations:** 1 Institute of Clinical Genetics and Tumor Genetics, Bonn, Germany; 2 Leibniz Institute for Analytical Sciences (ISAS), Dortmund, Germany; 3 John Walton Muscular Dystrophy Research Centre, Institute of Genetic Medicine, Newcastle University, Newcastle upon Tyne, UK; 4 Department of Medical Biology, School of Medicine, Dokuz Eylul University, Izmir, Turkey; 5 Department of Clinical Neurosciences, University of Cambridge School of Clinical Medicine, Cambridge Biomedical Campus, Cambridge, UK; 6 Pediatric Neurology, Faculty of Medicine, University of Duisburg-Essen, University Hospital, Essen, Germany; 7 Children’s Hospital of Eastern Ontario Research Institute, University of Ottawa, Ottawa, ON Canada; 8 Division of Neurology, Department of Medicine, The Ottawa Hospital, Ottawa, ON Canada; 9 Brain and Mind Research Institute, University of Ottawa, Ottawa, Canada; 10 Centro Nacional de Análisis Genómico (CNAG-CRG), Center for Genomic Regulation, Barcelona Institute of Science and Technology (BIST), Barcelona, Catalonia Spain; 11 Izmir Biomedicine and Genome Center, Dokuz Eylul University Health Campus, Izmir, Turkey; 12 Izmir International Biomedicine and Genome Institute, Dokuz Eylul University, Izmir, Turkey

**Keywords:** Goltz syndrome, Focal dermal hypoplasia, Protein-serine O-palmitoleoyltransferase porcupine, Fibroblast proteomics, Lamin a/c, Connective tissue disorder, ER-stress

## Abstract

**Background:**

Goltz syndrome (GS) is a X-linked disorder defined by defects of mesodermal- and ectodermal-derived structures and caused by *PORCN* mutations. Features include striated skin-pigmentation, ocular and skeletal malformations and supernumerary or hypoplastic nipples. Generally, GS is associated with in utero lethality in males and most of the reported male patients show mosaicism (only three non-mosaic surviving males have been described so far). Also, precise descriptions of neurological deficits in GS are rare and less severe phenotypes might not only be caused by mosaicism but also by less pathogenic mutations suggesting the need of a molecular genetics and functional work-up of these rare variants.

**Results:**

We report two cases: one girl suffering from typical skin and skeletal abnormalities, developmental delay, microcephaly, thin corpus callosum, periventricular gliosis and drug-resistant epilepsy caused by a *PORCN* nonsense-mutation (c.283C > T, p.Arg95Ter). Presence of these combined neurological features indicates that CNS-vulnerability might be a guiding symptom in the diagnosis of GS patients. The other patient is a boy with a supernumerary nipple and skeletal anomalies but also, developmental delay, microcephaly, cerebral atrophy with delayed myelination and drug-resistant epilepsy as predominant features. Skin abnormalities were not observed. Genotyping revealed a novel *PORCN* missense-mutation (c.847G > C, p.Asp283His) absent in the Genome Aggregation Database (gnomAD) but also identified in his asymptomatic mother. Given that non-random X-chromosome inactivation was excluded in the mother, fibroblasts of the index had been analyzed for PORCN protein-abundance and -distribution, vulnerability against additional ER-stress burden as well as for protein secretion revealing changes.

**Conclusions:**

Our combined findings may suggest incomplete penetrance for the p.Asp283His variant and provide novel insights into the molecular etiology of GS by adding impaired ER-function and altered protein secretion to the list of pathophysiological processes resulting in the clinical manifestation of GS.

**Supplementary Information:**

The online version contains supplementary material available at 10.1186/s13023-021-02068-w.

## Background

Goltz syndrome (GS, OMIM #305600), also known as focal dermal hypoplasia (FDH) is a X-linked (Xp11.23) dominant multi-systemic disorder first described by Robert W. Goltz in 1962 [1]. In 2007, clinical manifestation of GS was linked to mutations in *PORCN* [2, 3] encoding a 461 amino acid sized (52 kDa) protein named porcupine located to the Endoplasmic Reticulum (ER) and known to be involved in processing, secretion and signaling of Wnt proteins [4–6]. Wnt proteins are critical for interactions between ectoderm and mesoderm during embryogenesis [7–11]. Of note, mutations of *PORCN* are also associated with the pentalogy of Cantrell and the limb-body wall complex [12].

A total of 257 different public variants are registered in the online *PORCN* mutation database (LOVD, http://www.lovd.nl/porcn, accessed on August 4, 2020). *PORCN-*variants are distributed throughout the coding sequence with exception of exon 7. A total of 219 variants are predicted to be pathogenic based either on the nature of the change or the location in a functional domain; 19 variants are defined as benign and 19 have been classified as variant of uncertain significance (VUS), respectively. Mutations vary considerably: nonsense variants are the most common (n = 101), followed by missense (n = 95) changes. 38 frameshift and 28 splicing variants are described. Twelve patients have been published with large deletions in *PORCN*, 11 cases present with deletions of the whole gene [13].

The GS-phenotype exhibits a wide range of clinical findings: very characteristic features are skin abnormalities including patchy skin aplasia, subcutaneous fat herniation, papilloma, telangiectasia, sparse hair, dysplastic nails and linear hypo-/hyperpigmentation. Additionally, skeletal malformations, such as syndactyly, ectrodactyly, oligodactyly, transverse limb and long bone reduction defects are commonly described. Ocular malformations include anophtalmos, microphthalmia, cataract and choroidal retinal coloboma. Moreover, craniofacial dysmorphisms, such as a facial asymmetry, notched nasal alae and a pointed chin as well as abnormal ear morphology and dental defects are described [14].

To date only a few published case reports of GS, describing abnormalities of the central nervous system such as spina bifida and myelomeningocele, epilepsy, microcephaly, Arnold-Chiari malformation, hydrocephalus, agenesis of corpus callosum and cerebellar dysplasia (Table 1) [15–18] have been published. Besides, intellectually disability occurred only in some patients [16, 18–21].

**Table 1:** Neurological findings in *PORCN*-patients

Patient (reference)	(1) Bornholdt et al. [22]	(2) Vreeburg et al. [24]	(3) Kanemura et al. [18]	(4) Peters et al. [15]	(5) Brady et al. [34]	(6) Case reported girl	(7) Case reported boy	Frequency
Sex	M	M	F	M	M	F	M	5/7
Spina bifida	NR	−	+	−	−	NR	NR	1/7
Myelomeningocele	NR	−	−	+	NR	NR	NR	1/7
Epilepsy	+	−	+	−	NR	+	+	4/7
Microcephaly	+	−	+	+	+	+	+	6/7
Arnold-Chiari malformation	NR	−	−	+	NR	−	−	1/7
Hydrocephalus	NR	−	−	+	NR	−	−	1/7
Agenesis corpus callosum	NR	−	−	+	NR	−	−	1/7
Thin corpus callosum with periventricular gliosis	−	−	−	−	−	+	−	1/7
Cerebellar dysplasia	−	−	+	−	−	−	−	1/7
Cerebral atrophy with myelination delay	−	−	−	−	−	−	+	1/7
Intellectual disability	NR	NR	+	NR	NR	NR	NR	1/7
Developmental delay	NR	NR	+	NR	NR	+	+	3/7

GS is much more common in females as germline mutations in hemizygous males generally result in embryonic lethality. Only a small number of mosaic male cases have been reported (Additional file 1: Table S1) [2, 14, 15, 22–29], caused either due to de novo post-zygotic mutations occurring during development or in conjunction with a Klinefelter (XXY) karyotype.

## Results

### Molecular genetic findings

**Fig. 1:**
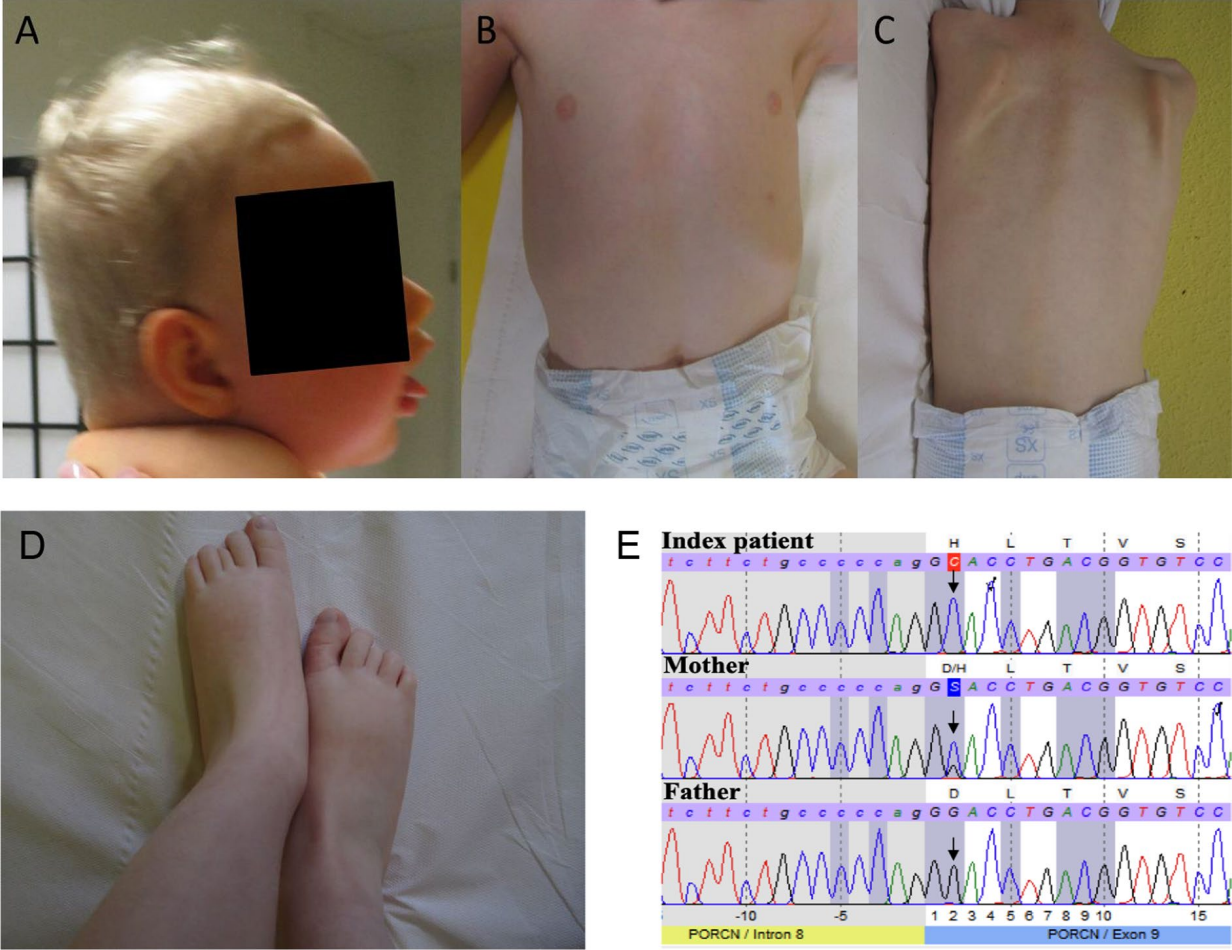
Clinical findings in patient 1 and molecular genetic findings in the family of patient 1: **A** triangular face, broad forehead, protruding eyebrow arches, narrow chin, full cheeks and lips, large ears and plagiocephaly. **B** Chest with supernumerary nipple. **C** Dorsal skin without pigmental or structural anomalies. **D** Feet with partial syndactyly D II/III and brachydaktyly DIII. **E** Electropherogram of Sanger sequencing reveals for patient 1 point mutations at position c.847G > C (p.Asp283His) in exon 9 of the *PORCN* gene. The mother shows a wildtype and a mutant signal. The father indicates only a wildtype signal (indicated by the arrows)

By whole exome sequencing of patient 1, we identified a novel hemizygous missense variant p.Asp283His (hg19:chrX:48372914; c.847G > C, p.(Asp283His); CCDS14299, NM_203475) in *PORCN* in a patient 1. The variant is absent in ExaC, predicted to be tolerated by SIFT but to be damaging by Polyphen2 and Mutation**-**Taster. The CADD score is 23.7. Subsequent segregation studies by Sanger sequencing showed a non-mosaicism in the index case and revealed the presence of the same heterozygous variant in the healthy mother (Fig. 1). Based on the findings of the segregation analyses, studies of X-chromosome inactivation were performed in the mother. These studies did not show a skewed inactivation (data not shown). Given that the G > C variant is located in proximity to the acceptor splice site, RNA sequencing studies were performed to rule out the occurrence of splice abnormalities in terms of the creation a leaky splice site or the generation different splice isoforms, in turn leading to parallel expression of amounts of wildtype and mutant protein. These studies focused on amplicons spanning exon 4–13 and revealed no differences between patient-derived and control cDNA (data not shown).

In patient 2, trio exome sequencing revealed a heterozygous de novo nonsense variant p.Arg95Ter (hg19: X:48369829; c.283C > T (p.Arg95Ter); ENST00000326194) in *PORCN,* previously reported in GS patient. The variant is absent in ExAC/-gnomAD and has a CADD score of 35.

### Comparison of clinical neurological features and male phenotypes reported elsewhere and the findings of our patients

Clinical features for both sexes include striated skin-pigmentation and ocular and skeletal malformations. Abnormalities of the central nervous system have only been described scarcely in GS patients (Table 1). Most detected CNS-features are microcephaly (6/7) followed by developmental delay (3/7). Intellectual disability (1/7), spina bifida (1/7) and hydrocephalus (1/7) occurred only in single patients presenting with further neurological symptoms. Our two cases present with epilepsy and some of the previous reports describe GS as a seizure disorder: out of 7 patients summarized in Table 1, 4 individuals (2 males and 2 females) were affected by brain malformations.

Mostly, GS in males is associated with in utero lethality. Reported male patients showed mosaicism due to de novo post-zygotic mutations in the *PORCN* gene and to date there are only three cases of non-mosaic surviving males with inherited mutations described in literature (Additional file 1: Table S1). Most of the male patients show ectodermal (skin (26/31), mesodermal (syndactyly (25/31) and ectrodactyly (17/31)) features as well as ocular malformations (microphthalmia (9/31), coloboma (11/31) and other ocular defects (13/31)). Brain malformations occur in 8 of 31 male patients. All non-mosaic surviving males have brain defects, but typical striatic skin abnormalities are not observed. This accords with the clinical presentation of our patient and suggests that non-mosaic mutations might lead to “GS-sub-phenotypes” compatible with life.

### Expression of p.Asp283His mutant PORCN alters protein secretion of skin fibroblasts

**Fig. 2:**
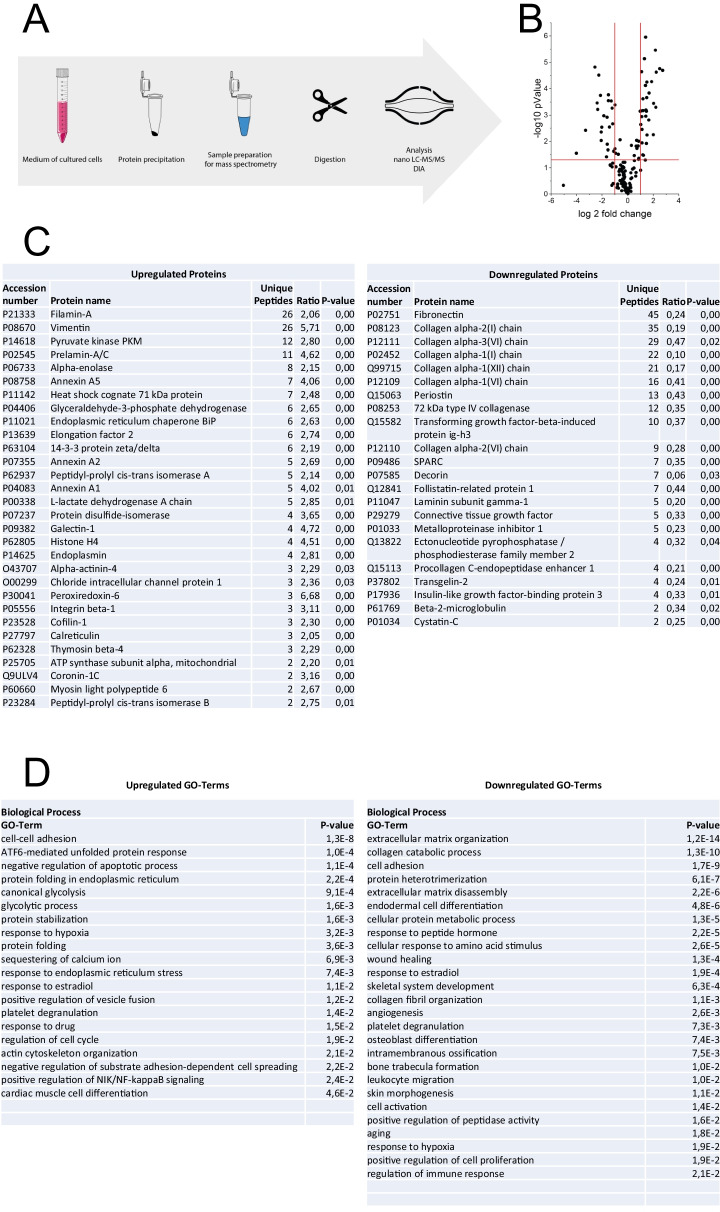

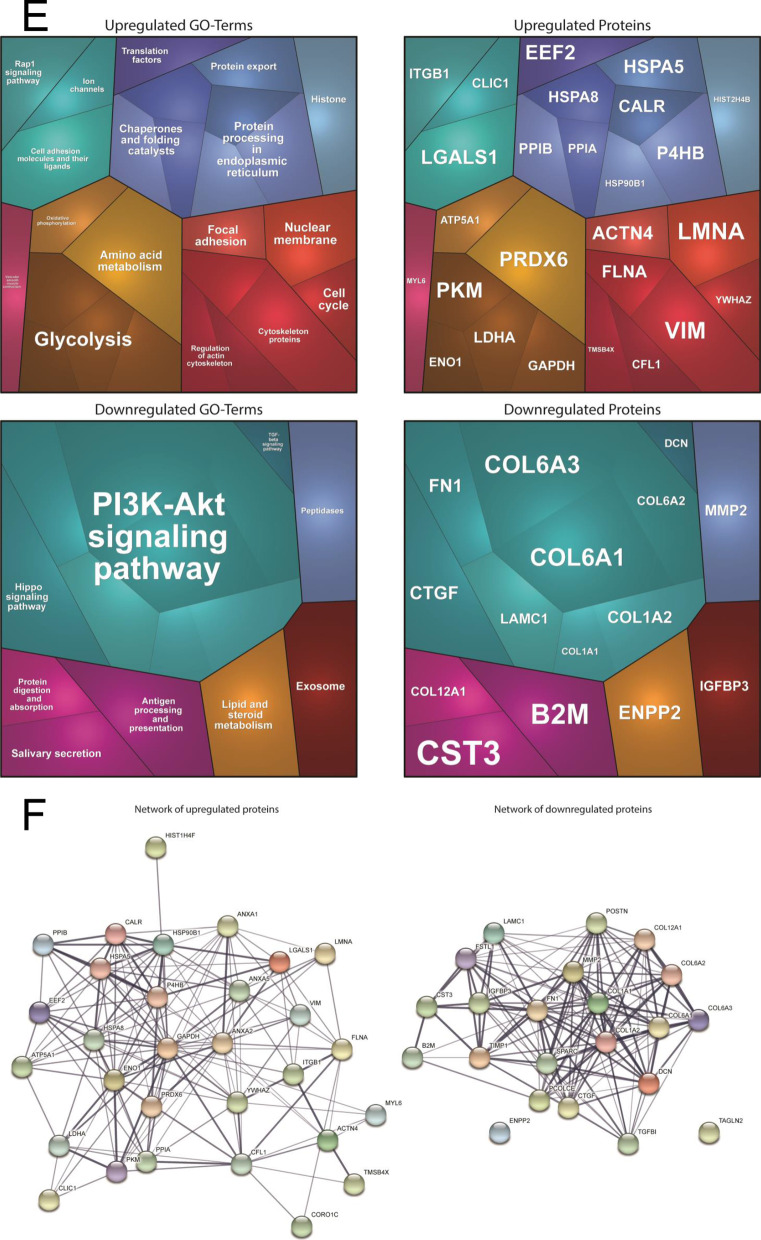
Proteomic studies: **A** Scheme of the workflow. **B** Volcano plot displaying fold of changes of dysregulated (up- and down-regulations) proteins. **C** List of up- and downregulate proteins. **D** List of biological processes affected by up- and down-regulation of the different protein classes. **E** Proteomaps-based in silico analysis of cellular functions affected by the respective dysregulated proteins. **F** STRING-based analysis of functional interaction of dysregulated proteins

Comparative mass spectrometric analysis of culture media from triplicates of pulse-chased *PORCN*-patient derived versus control skin fibroblasts (gender- and age-matched) was performed to determine secretomic changes caused by expression of p.Asp283His mutant PORCN. This approach was already successfully applied to unravel secretion defects in a synaptic transmission disorder [30] and was prompted by the fact that the ER-resident PORCN modulates processing and secretion of Wnt-related proteins [31]. The comparative mass spectrometric analysis presented in this study is based on an excellent coverage of the experimentally assessable human skin fibroblast proteome (96,512 peptides referring to 8280 proteins spanning five order of magnitude: [32] (Fig. 2). This experiment revealed increased secretion of 30 proteins whereas 22 proteins were less secreted (Fig. 2). In line with the known subcellular localization of PORCN [31], ER-resident proteins known to be controlled by ER-stress such as BiP (GRP78), Endoplasmin (GRP94), Protein disulfide-isomerase (PDI), Heat shock cognate 71 kDa protein, Peptidyl-prolyl cis–trans isomerase B and Calreticulin are increased in the secretome (Fig. 2). This suggests an altered cellular abundance of these chaperones. In addition, structural proteins such as Filamin A, Vimentin, Alpha-actinin-4, Thymosin beta-4 and Myosin light polypeptide 6 are more abundant in the secretome of patients derived cells (Fig. 2). Dysregulated Annexins (A1, A2, A5) are known to promote rearrangement of the actin cytoskeleton (Fig. 2). A general profound decrease of Collagens, 72 kDa type IV collagenase and Procollagen C-endopeptidase enhancer 1 was identified in the secretome of PORCN-patient fibroblasts. This is accompanied by decrease of other proteins crucial for composition of the extracellular matrix such as Periostin, Decorin, Fibronectin, Connective tissue growth factor, Follistatin-related protein 1, SPARC and Laminin subunit gamma-1 (Fig. 2). In addition, Prelamin-A/C is more abundant in the secretome of patients derived cells (Fig. 2).

GO-term based analysis of biological processes affected by the expression of p.Asp283His mutant PORCN in fibroblasts was performed. For proteins increased in the secretome, this in silico approach also suggested altered protein processing, ER-stress and cytoskeleton. Furthermore, altered glucose metabolism, calcium handling, cell-adhesion, -spreading and -differentiation as well as NF-kappaB signaling are indicated (Fig. 2). Regarding the predicted biological processes affected by proteins decreased in the secretome, apart from ECM composition impacting on cell adhesion and (presumably) cell differentiation, altered angiogenesis, skin morphogenesis and skeletal system development are suggested (Fig. 2). Further in silico studies toward the identification of altered cellular pathways were performed based on proteomaps (see above). For proteins with increased secretomic abundance, this approach confirmed perturbed protein processing, cytoskeleton, and glucose metabolism as well as (based on increased secretion of Prelamin-A/C) suggested an impact on nuclear envelope integrity (Fig. 2). For proteins with decreased abundance in the secretome, proteomaps-based in silico studies (based on the dysregulation of ECM proteins) hint toward perturbed signalling pathways including PI3K-AKT-, TGF-beta- and Hippo-signalling (Fig. 2). To unravel functional protein association networks, a STRING network analysis was performed for increased and decreased proteins, respectively. Of note, for all increased proteins, a functional interplay was identified whereas for decreased proteins only two (ENPP2: Ectonucleotide pyrophosphatase/phosphodiesterase family member 2 and TAGLN2: Transgelin-2) are not functionally connected with the others (Fig. 2). Hence, a tight functional connection of all dysregulated proteins can be postulated.

### Expression of p.Asp283His mutant PORCN alters protein composition and distribution in skin fibroblasts and impacts on cellular fitness and protein clearance

**Fig. 3:**
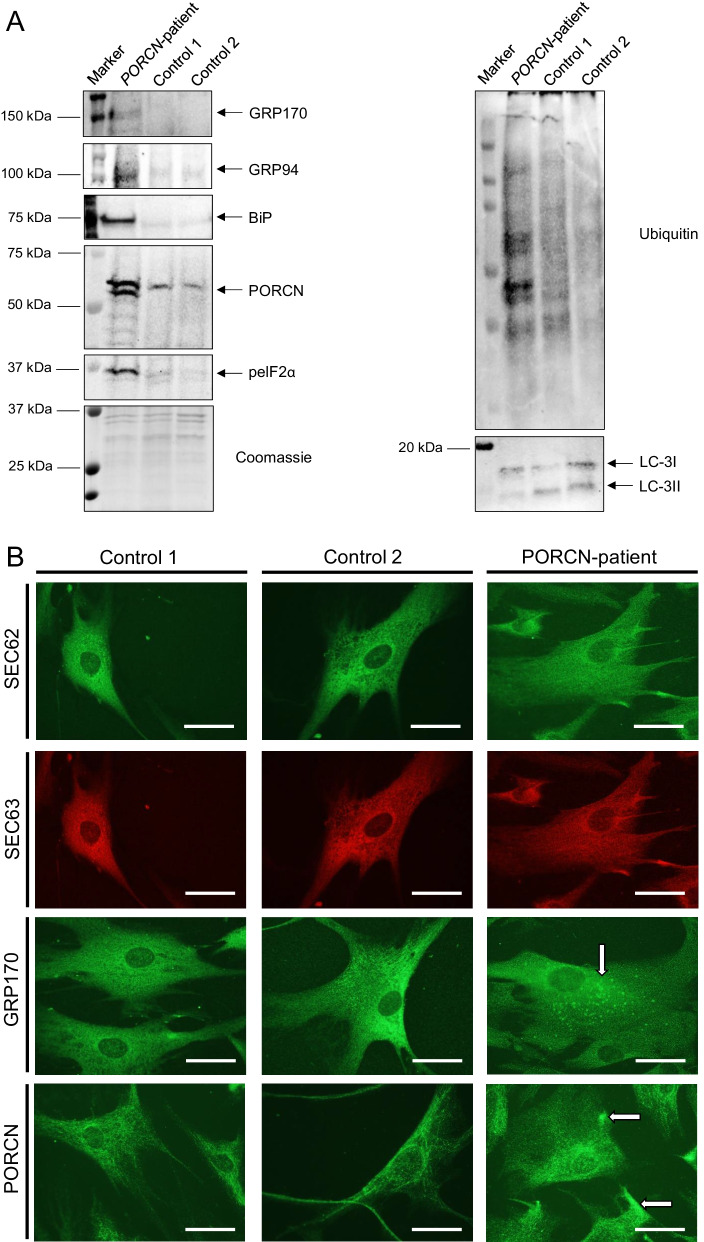

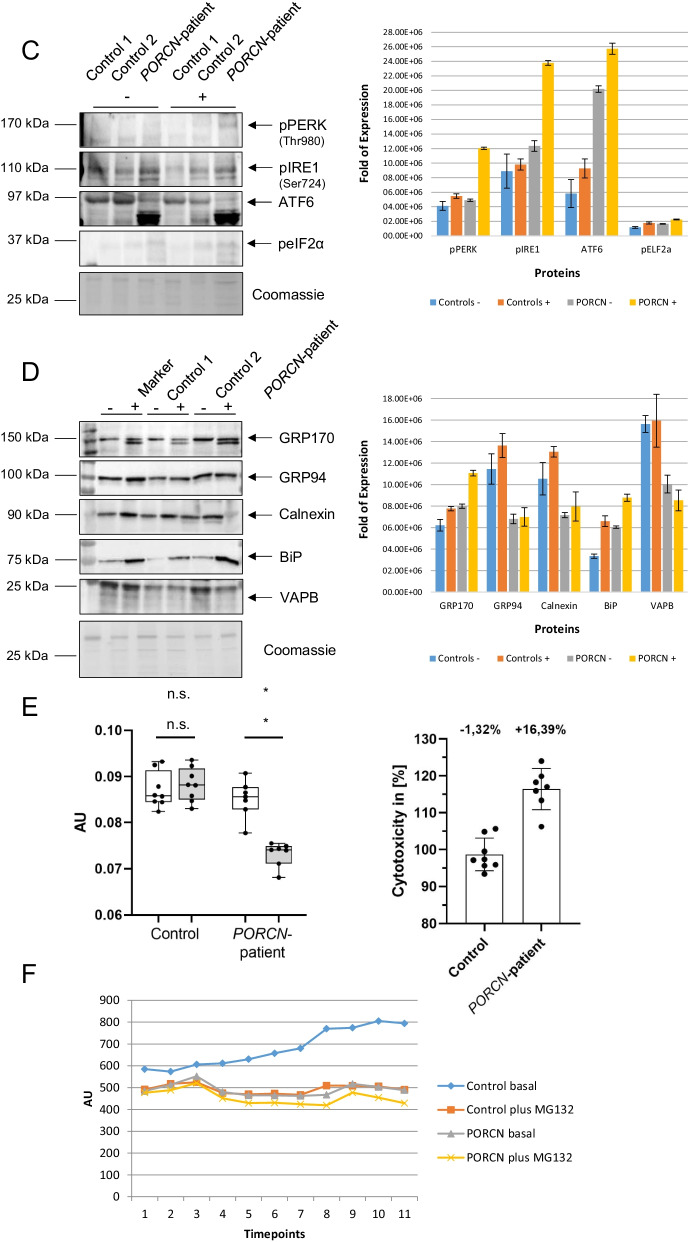
Immunological studies of ER-homeostasis and protein clearance on fibroblasts: **A** Immunoblot studies revealed increase level of GRP170, GRP94, BiP and peIF2a in protein extracts derived from the PORCN-patient compared to controls. Immunoblot studies of PORCN revealed an increase along with the presence of an additional band of lower molecular weight compared to the analysis of the PORCN protein in control fibroblasts. Additionally, immunoblot studies of protein-ubiquitination revealed an increase in patient-derived fibroblasts along with a decrease of LC3-I to LC3-II conversion. Coomassie blue staining was performed to demonstrate equal protein loading. **B** Immunofluorescence studies of SEC62, SEC63, two ER-membrane resident proteins, revealed a blurred immunoreactivity in fibroblasts derived from the patient compared to the respective protein distributions observed on control cells. Immunofluorescence of GRP170 revealed a cytoplasmic punctuate accumulation in patient-derived fibroblasts (white arrow) compared to the reticular protein distribution detected in control cells. Immunofluorescence studies of PORCN showed focal cytoplasmic accumulations in patient-derived cells (white arrows) compared to the observed immunoreactivity in control cells. Scale bars = 30 µm. **C** Immunoblot-based investigation of ER-stress response of Tunicamycin treatment (− = without treatment, +  = with treatment) focussing on the three major transducers of the UPR revealed increased phosphorylation of PERK and IRE1 in patient-derived fibroblasts whereby on PERK-phosphorylation was enhanced after Tunicamycin-treatment. Along this line, increased proteolytic cleavage of ATF6 toward its activation is visible in patient-derived cells. This effect is not significantly further pronounced in patient-derived cells after Tunicamycin-treatment. Controls were merged in the diagrams. **D** Immunoblot-based investigation of ER-stress response of Tunicamycin-treatment (− = without treatment, +  = with treatment) focussing on down-stream factors revealed a more pronounced increase of BiP and GRP170 in patient-derived cells in treated cells. Whereas GRP94 showed an increased in stressed control fibroblasts, patient-derived cells did not present with elevated level after stressing. Along this line, increase of Calnexin in stressed patient fibroblasts was less pronounced compared to control cells. In stressed patient-derived fibroblasts VAPB-level were decreased whereas in control cells level remained unchanged after Tunicamycin-application. Controls were merged in the diagrams. **E** Results of MTT-assay based studies focussing on cellular metabolic activity. Left panel visualizes the metabolic activity of control and patient-derived cells under basal conditions (white box plots) and after Tunicamycin-treatment (grey box plots). AU: arbitury unit. Right panel: Tunicamycin-treatment results in a slight decrease of cytotoxicity in control fibroblasts (most likely based on the activation of compensatory mechanisms) whereas a profound increase of cytotoxicity is detectable in PORCN-patient derived cells. **F** Studies of proteasomal activity in control (blue and orange) and patient-derived (grey and yellow) cells reveal an activity-decrease under basal conditions. In comparison to control cells, patient-derived cells do not show a fundamental decrease of this activity after MG132-treatment. Y-axis: 350/440 nm; X-axis: time points in 5 min intervals between measurements

To investigate if the expression of p.Asp283His mutant PORCN correlates with presence of ER-stress and activation of the unfolded protein response (UPR), we studied peIF2α (eIF2α was increased in the secretome), Calreticulin, GRP94, BiP and GRP170 (a nucleotide exchange factor of BiP) by immunoblot analyses. Apart from increased phosphorylation of eIF2α, increased level of the above-mentioned chaperones was identified along with increased ubiquitination of proteins and decreased conversion of LC3-I to LC3-II (Fig. 3A, B) suggesting UPR activation and perturbed protein-clearance in patient-derived fibroblasts. We next investigated ER-morphology by focussing on the distribution of SEC62 and SEC63, two integral ER-membrane proteins, as well as GRP170: whereas in fibroblasts derived from control individuals, a reticular staining for these three proteins was detected, fibroblasts derived from the patient with *PORCN* variant presented with a more diffuse immunoreactivity. For GRP170 the presence of punctate accumulations was observed (Fig. 3B). These results prompted us to next investigate if this perturbed ER-homeostasis leads to a broad UPR-activation. Indeed, immunoblot studies of the three major UPR-transducers, pPERK, pIRE1 and ATF6, indicated an activation of the three UPR-branches by enhanced phosphorylation of PERK and IRE1 as well as by enhanced proteolytic cleavage of ATF6, respectively (Fig. 3C). Only PERK-phosphorylation was enhanced by Tunicamycin-treatment (triggering ER-stress) (Fig. 3C). However, we hypothesized that additional stress burden might impact on downstream-factors of the three UPR-branches. To investigate this assumption, again fibroblasts were exposed for 16 h to Tunicamycin and afterwards immunoblot studies on whole protein extracts were carried out: focussing on changes in expression of BiP along with its co-chaperone GRP170 and on GRP94, Calnexin and VAPB. A more pronounced increase in patient-derived cells was identified for BiP and GRP170 suggestive for an increased ER-vulnerability against additional stress burden. In contrast, whereas GRP94 showed an increased in stressed control fibroblasts, patient-derived cells did not present with elevated level after stressing suggesting a defect in the broad activation or a selective activation of ER-related chaperones modulating UPR. Increase of Calnexin in stressed patient fibroblasts was less pronounced compared to control cells. In stressed patient-derived fibroblasts, VAPB-level were decreased whereas in control cells level remained unchanged after application of the ER-stressor (Fig. 3D). Experiments were performed thrice with similar results.

We next investigated the fitness of the fibroblasts by applying an MTT-assay under basal conditions and after Tunicamycin-treatment (eight biological replicates): fibroblasts derived from the PORCN-patient per se presented with reduced cellular metabolic activity as an indicator of cell viability and proliferation compared to fibroblasts derived from an age- and sex-matched controls (Fig. 3E). Whereas Tunicamycin-treatment resulted in a slight increase of metabolic activity in control cells (most likely based on the successful activation of cellular defence/rescue mechanisms), this was significantly reduced in PORCN-patient derived cells (Fig. 3E, left panel). This functional observation accords with a minor decrease of cytotoxicity (1.32%) in Tunicamycin-treated control fibroblasts whereas patient-derived fibroblasts present an 16.39%-fold increase of cytotoxicity after treatment (Fig. 3E, right panel).

Based on increase of ubiquitinated proteins and decreased LC-3I to LC-3II conversion in PORCN-mutant fibroblasts, proteasomal activity was studied revealing a significant decrease in the patient cells compared to the controls (Δ306 after 55 min of measurement in 5 min intervals; Fig. 3F). However, Tunicamycin-treatment did not result in a more profound impaired function in patient cells compared to control fibroblasts (Δ62 after 55 min of measurement in 5 min intervals; Fig. 3F).

**Fig. 4:**
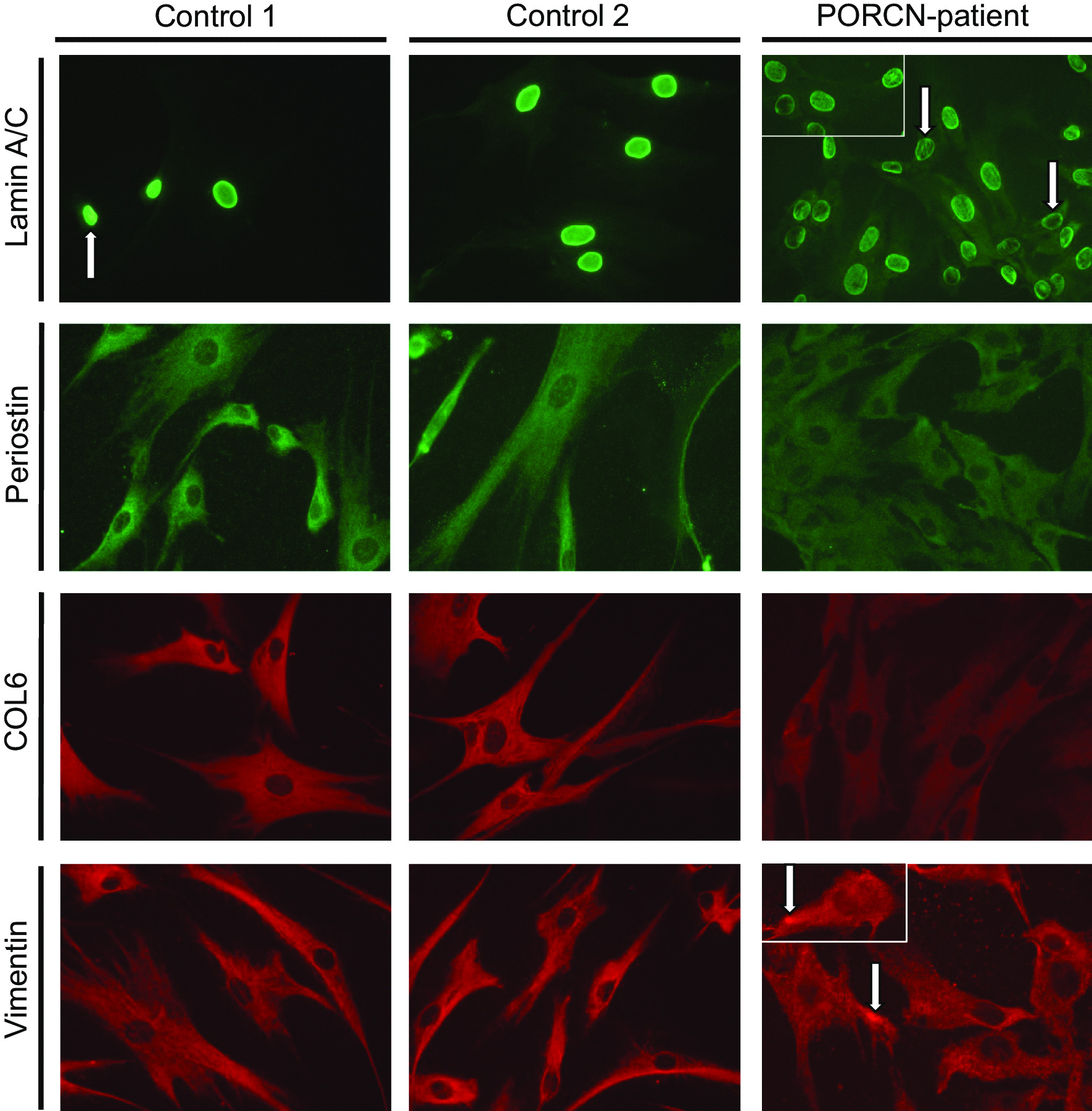
Immunological studies to confirm proteomic data: Immunofluorescence studies revealed the presence of irregular nucleoplasmic Lamin A/C-depositions (often appearing as rods) in cells derived from the patient in addition to nuclei with remarkable decreased immunoreactivity (white arrows). Periostin and Collagen alpha-3 (VI) decreased cellular level in patient-derived cells. Vimentin staining revealed a uniform distribution throughout the cytoplasm of control fibroblasts, in patient-derived cells, the fluorescence intensity of reticular Vimentin staining is less intense. Moreover, focal increase of Vimentin within the cytoplasm and adjacent to the plasma membrane was frequently identified in patient-derived fibroblasts. Scale bars = 30 µm

To confirm the proteomic findings and to investigate if altered secretion correlates with cellular changes in protein abundances, paradigmatic proteins were investigated by immunofluorescence: Lamin A/C-immunolabelling (prompted by the increased secretion of Prelamin-A/C) frequently revealed the presence of irregular nucleoplasmic depositions (often appearing as rods) in cells derived from the patient in addition to nuclei with remarkable decreased immunoreactivity (Fig. 4 white arrows). Focussing on ECM-related proteins, Periostin and Collagen alpha-3(VI) were studied and (in line with the secretomic findings) decreased cellular level of both were identified in patient-derived cells suggesting a defect in cellular production of ECM proteins (Fig. 4). While Vimentin staining revealed a uniform distribution throughout the cytoplasm of control fibroblasts, in patient-derived cells, the fluorescence intensity of reticular Vimentin staining is less intense. However, focal increase of Vimentin within the cytoplasm and adjacent to the plasma membrane was frequently identified in patient-derived fibroblasts (Fig. 4).

## Discussion

### Clinical findings

A literature review was performed to summarize CNS features described in *PORCN*-patients and revealed that microcephaly (6/7) represents the most common CNS feature followed by epilepsy (4/7), developmental delay (3/7) while intellectual disability (1/7), spina bifida (1/7) and hydrocephalus (1/7) occur only in individual cases. Seizures were also present in our two cases and previous reports describe GS as a seizure disorder (Table 1). Of note, a previous study defined additional roles for PORCN in controlling synaptic transmission by regulating the level and composition of hippocampal AMPA receptor (AMPAR) complexes which mediate most fast excitatory neurotransmission in the brain [33].

In contrast to the currently known clinical spectrum associated with the p.Arg95Ter mutation including linear skin lesions, asymmetrical skeletal defects, absence deformities of bones, clinodactyly, dental defects and microcephaly [22], our patient 2 also presents epilepsy. This indicates that no clear genotype–phenotype correlation exists.

A further literature review of GS cases showing seizures revealed also no correlation between the exonic localization of the mutation and the manifestation of seizures (BORNHOLDT et al*.* 2009: exon 14, KANEMURA et al*.* 2011: exon number in NM_203475.3, patient 1: exon 10, patient 2: exon 3) [18, 22].

In line with the clinical observations described by [34] and [35] for boys carrying *PORCN* mutations in non-mosaicism, also our patient 1 shows brain abnormalities and does not present profound skin perturbations. This finding suggests that non-mosaicism *PORCN* mutations in males might lead to a GS-sub-phenotype defined by the presence of CNS-perturbations and the absence of skin anomalies. Further functional and biochemical studies on patient-derived cells (such as fibroblasts) are needed to unravel why male mosaic patients more frequently present with skin anomalies. However, one might speculate that this phenomenon is caused by the activation of compensatory mechanisms in fibroblasts.

The p.Asp283His mutant form of *PORCN* was found in the asymptomatic mother and the possibility of a skewed X-chromosome inactivation potentially leading to the discordant phenotypes was excluded. Nevertheless, results of our further biochemical studies suggested an effect of the expression of p.Asp283His-mutant PORCN and cellular proteostasis (see below). One might speculate that different aspects including epigenetics, expression of modifiers or even the presence of further mutations affecting other proteins crucial for proper ER-function and/ or Wnt signaling might lead to the incomplete penetrance of the p.Asp283His amino acid substitution.

### Pathogenicity of identified PORCN variants

Whereas the heterozygous de novo nonsense variant p.Arg95Ter identified in patient 2 fits with the clinical presentation of the girl and had been previously described to be pathogenic [22], the p.Asp283His amino acid substitution identified in patient 1 was inherited from the healthy mother who showed no sign of a non-random X-chromosome inactivation. Moreover, transcript sequencing studies were carried out to address the question if the G > C substitution alters splicing and thus leads to the reduced abundance of full-length porcupine that drives the phenotype—which could perhaps explain why the mother is asymptomatic, and even also why a second band was observed by immunoblot studies. However, these studies did not reveal the occurrence of altered transcript whereby the presence of the point mutation could be confirmed.

This prompted us to address the pathogenicity of the p.Asp283His-PORCN expression at biochemical level: by utilizing patient-derived skin fibroblasts, first we focused on the effect of the amino acid substitution on the properties of PORCN. While immunoblotting studies revealed an additional band for the protein compared to whole protein extracts of control fibroblasts, immunofluorescence studies showed a diffuse staining in patient derived cells derived from the patient but a reticular network in control fibroblasts. Both findings indicate a pathogenic effect of p.Asp283His-PORCN expression. This assumption is additionally supported by the immunofluorescence studies revealing a more diffuse immunoreactivity of SEC62 and SEC63, two components of the ER, and a punctate accumulation of GRP170. Additionally, immunoblotting studies on whole protein extracts of Tunicamycin-exposed fibroblasts revealed that the patient derived cells were more sensitive. Given that PORCN is known to modulate the processing of Wnt-proteins within the ER, the secretome of fibroblasts expressing p.Asp283His mutant PORCN was compared to the ones of control cells by untargeted proteomic profiling. This experiment showed altered protein secretion and—in line with the subcellular localization of PORCN—suggested the presence of ER-stress and UPR activation by the three major transducers. Immunological-based studies confirmed this assumption and thus provided further evidence for a pathogenic effect of p.Asp283His mutant PORCN expression. However, the inclusion of additional fibroblasts samples (which were not available for our studies) would have allowed to dissect the effects specific to the p.(Asp283His) in comparison other PORCN-missense variants, if any.

Significant reduction of proliferation of PORCN-patient derived cells along with increased cytotoxicity after Tunicamycin-treatment indicates a higher sensitivity of the ER expressing mutant PORCN compared to control cells thus supporting the results of our immunoblot studies. To study if the perturbed ER-homeostasis associated with increase of ubiquitinated proteins impacts on the protein clearance machinery, proteasomal activity was investigated under basal and Tunicamycin-stressed conditions. Interestingly, proteasomal activity was reduced in patient derived cells compared to controls under basal conditions but Tunicamycin-treatment did not result in profound differences between patient and control fibroblasts. This finding along with the decreased LC-3I to LC-3II conversion as identified by our immunoblot studies suggests a perturbed protein clearance in PORCN-patient derived cells. However, further biochemical studies are needed for a precise molecular characterization of perturbed protein clearance in PORCN-mutant cells.

Wnt pathways have been proposed to regulate cell polarity and migration based on cytoskeletal remodeling, and relationships with cell–cell adhesion both impacting on cilia/ciliogenesis [36, 37]. Results of our biochemical studies indicated perturbed cytoskeleton in fibroblasts of the PORCN-patient by highlighting that cytoskeletal proteins are more secreted and that Vimentin, a class-III intermediate filament, shows an altered cellular distribution with focal accumulations adjacent to the plasma membrane and cellular extensions. Given that Vimentin is attached to the nucleus and ER, a functional continuum connected by the nuclear envelope, the coincident finding of irregular nuclear distribution of Lamin A/C not only further supports the concept of a pathogenic impact of expression of p.Asp283His mutant PORCN but also suggests that affection of cytoskeletal and nuclear envelope proteins along with ER-stress might represent a pathophysiological interplay arising from altered Wnt signaling. Regarding the impact of Wnt signaling on cell–cell adhesion [36, 37], it is important to note that our combined findings suggest impaired production of this protein-class, a molecular observation in turn suggesting a pathogenic effect of the p.Asp283His mutation. This hypothesis is supported by the link between Wnt signaling and deposition of Collagen in fibroblast-like cells [38], further discussed below).

### Pathophysiological relevance of biochemical findings

Nascent Wnt polypeptides are targeted to the ER-lumen by signal peptides. As they are targeted to transit through the secretory pathway, there they undergo a series of modifications and thus associate with several proteins, including PORCN [39]. Perturbed protein processing within the ER leads to the activation of adaptive programmes such as the UPR aiming to improve protein folding and to promote quality control mechanisms [40]. UPR-activation with concomitant BiP increase was already shown in the context of perturbed Wnt signalling [41]. In fibroblasts derived from patient 1, we showed that the hemizygous p.Asp283His mutation results in expression of a regular sized PORCN in addition to an abnormal form. This was associated with elevated secretion of UPR-proteins including BiP (see above). Immunofluorescence studies showed increased abundance of UPR-proteins in patient-derived fibroblasts suggesting UPR-activation upon expression of mutant PORCN. This suggests a pathogenicity of the p.Asp283His mutation and unravels perturbed ER-homeostasis as part of the pathophysiology. This assumption represents an important aspect as the pathology of many disorders including neurological diseases includes accumulation of misfolded proteins. Strategies to target specific components of the UPR using small molecules and gene therapy are developed and promise interesting avenues for future interventions [42].

Secretion of Peptidyl-prolyl cis–trans isomerases occurs in response to oxidative stress (which shows a mutual crosstalk to ER-stress) through a vesicular secretory pathway that involves actin remodeling and myosin activation. Elevated secretion of this protein identified in PORCN-patient derived fibroblasts might correlate (i) with oxidative stress as indicated by concomitant increased secretion of Peroxiredoxin-6 and (ii) with cytoskeletal changes which are indicated by the results of our biochemical studies. Cytoskeletal changes were already associated with other multisystemic diseases originating from altered ER-related protein-processing capacity [43] and have an impact in the ethology of different disorders including neurological diseases [30, 44]. As for impaired protein processing avenues, also based on the known interaction of cytoskeletal proteins with small molecules [45], drugs targeting the cytoskeleton are currently being tested showing promising effects [46]. Hereby, compounds targeting signaling molecules which regulate cytoskeleton dynamics, constitute the mostly addressed therapeutic interventions [46]. Consequently, combination of drugs targeting both, the restoration of ER-homeostasis and proper cytoskeleton might represent a treatment strategy in PORCN-patients.

Wnt signaling in fibroblast-like cells activates axon re-growth via deposition of Collagen promoting spinal cord regeneration in zebrafish [38]. Thus, impaired production and secretion of Collagen proteins (as well as other proteins crucial for regular composition of the ECM) as identified in our study might represent a significant part of the pathophysiology impacting on the manifestation neurological findings. Although more functional studies are required to provide functional evidence for this assumption, the known impact of regular expression and distribution of ECM proteins in the genesis of skin and skeletal disorders [47, 48] suggests an impact of dysregulated ECM proteins in the overall pathology of the PORCN-related phenotypes. The fact that a variety of human syndromes are linked to defects in ECM protein secretion with respect to the landscape of biosynthetic and protein transport steps within the early secretory pathway [48] further supports this assumption. Hence, one might postulate that parts of the symptoms observed in PORCN-patients could be defined as a secondary connective tissue disorder arising from perturbed Wnt signalling and ER-function.

## Patients, materials and methods

### Clinical details

#### Patient 1

At the age of 20 months, a boy with developmental delay, microcephaly, brain malformation**s** and a drug-resistant epilepsy was seen for syndromal diagnostics. The family history was unremarkable. The patient is the first child of non-consanguineous healthy parents, both 32 years of age. The mother suffered from gestational diabetes during pregnancy that made a dietary treatment necessary. In the 37th of pregnancy, a small head was documented; the boy was born in gestational week 39 by vacuum extraction after a conspicuous cardiotocography (CTG). Birth parameters were all in the normal range. He weighed 3070 g and was 49 cm in length, his occipital frontal circumference was 33 cm (− 1.77 z). The first three well-baby exams showed no abnormalities. At an age of nine weeks seizures occurred for the first time. The drug treatment had been changed several times but was not successful. Multiple EEG studies were conspicuous. An underdevelopment of the brain substance was detected by MRI. MRI studies revealed atrophy of the cerebrum with a myelination delay. An ophthalmological, a hearing and a cardiac examination were all normal. A metabolic screening as well as a liquor analysis/CSF showed no pathological findings.

The physical examination at the age of 20 months was normal for length and weight but showed marked microcephaly with plagiocephaly. The boy weighed 11 kg, was 82.5 cm in length (− 0.7 z) and had a head circumference of 40 cm (− 7.4 z). Notably, nipples were wide-spaced with a supernumerary nipple below the left nipple (Fig. 1). The general muscle tone was increased. The skin was dry and had a pale color but otherwise inconspicuous. Noteworthy, the boy had also wide spaced teeth and delayed toothing (first deciduous tooth at the age of 12 months).

Skeletal abnormalities revealed a simian crease on the right palm-, pointed fingers with partial syndactyly (D II/III) and showed bilateral toe brachydactyly (D III). Abnormal facial gestalt of the patient included hypertelorism, a triangular face, a broad forehead, protruding orbital arches, a narrow chin, full cheeks and lips, large ears, and a flat nasal bridge (Fig. 1). Moreover, no visual contact was possible and there were also no deliberate eye movements. He had no autonomous head control and showed motor restlessness. There was no sign of speech development as babbling or willful sounding.

Molecular testing for Fragile-X syndrome, Coffin-Lowry syndrome, Pitt-Hopkins syndrome, Opitz BBB/G syndrome, and Mowat-Wilson syndrome was performed but revealed no pathological findings.

#### Patient 2

This patient, a 9 year old girl, presented with developmental delay and failure to thrive at 12 months of age. She was the first child of second cousin parents and born at full term via vaginal delivery (birth weight 3100 g (− 0.48 SDS), without any complications. In the neonatal period she was hospitalized due to poor feeding. At eight months of age she had surgery for developmental hip dysplasia. At 13 months of age, developmental assessment (Denver II test) showed severe delay in all four domains (gross motor at 1-month, fine motor at 2.5 months, language at 2.5 months, and personal-social skills at 1 month). She developed focal tonic and/or clonic seizures with impaired consciousness at three years of age. On electroencephalography (EEG) multifocal sharp waves and polyspikes, most evidently in the right central parietal region, were observed. The cranial magnetic resonance imaging revealed a thin corpus callosum and periventricular gliosis at the age of 10 months and seven years. She had drug-resistant epilepsy and was treated with oxcarbazepine, valproic acid and clonazepam. She received baclofen for spasticity. Percutaneous endoscopic gastrostomy tube was inserted due to failure to thrive at 8 years of age.

**Fig. 5:**
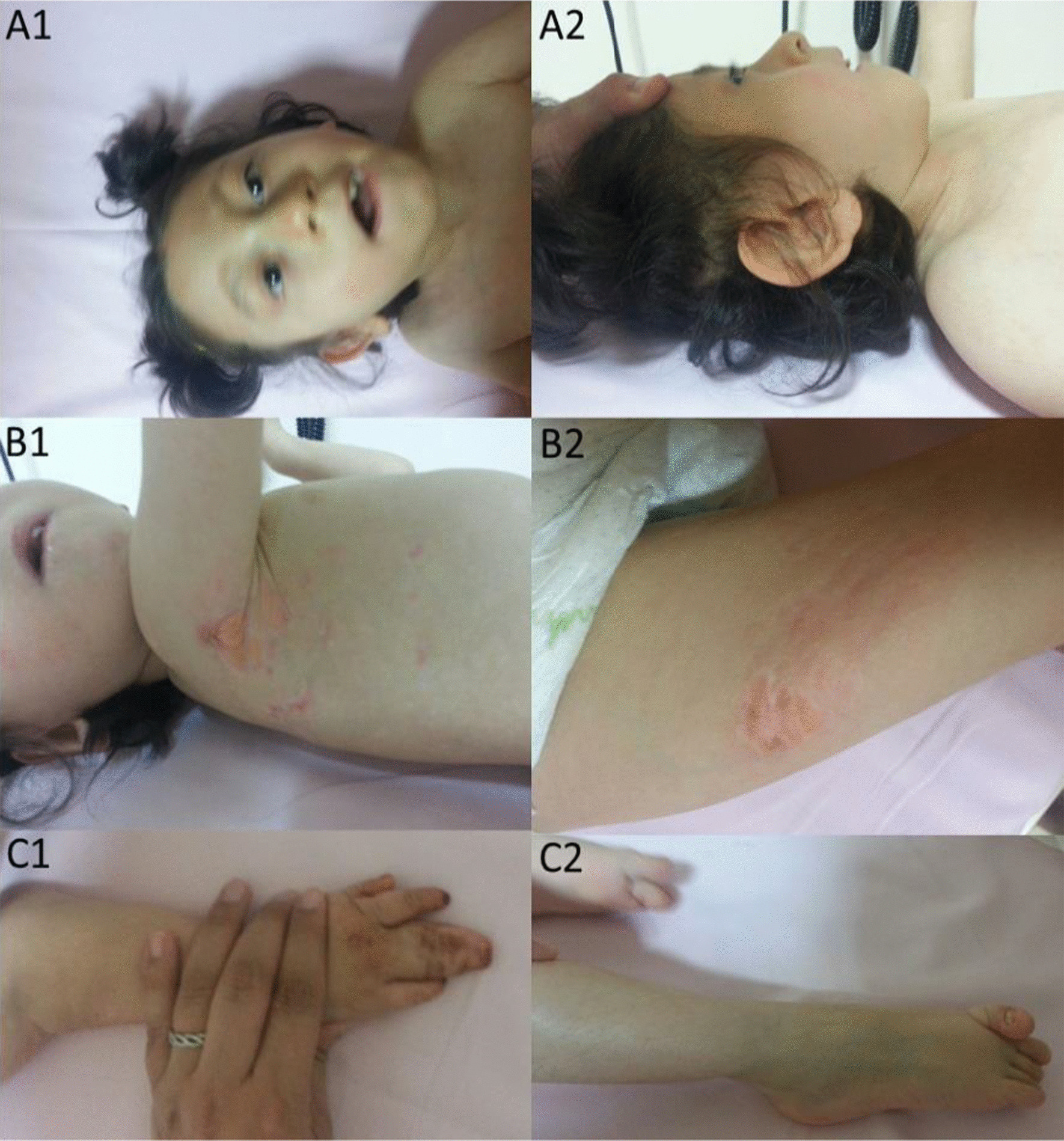
Clinical features of patient 2: **A1**, **A2** Facial features including microphthalmia, short neck, low hairline, highly arched eyebrows, hypoplastic helix, retrognathia, high arched palate, thin lips, dental abnormalities, and large ears. **B1**, **B2** Typical skin abnormalities. Atrophic erythematous linear lesions and focal herniations of subcutaneous tissue. **C1**, **C2** Skeletal malformations: include syndactyly of third and fourth fingers of the right hand, sacral dimple, pes planovalgus and overriding of toes

On physical examination her anthropometric measurements were recorded as weight 11 kg (− 6.1 SDS), height 90 cm (− 6.9 SDS), head circumference 42 cm (− 6.7 SDS). She did not have head control, eye contact or object tracking and was not able to sit unsupported. Her neurological examination revealed microcephaly, bilateral mild esotropia, axial hypotonia, spasticity and increased deep tendon reflexes in upper and lower extremities. She had dysmorphic facial features including microphthalmia, short neck, low hairline, highly arched eyebrows, hypoplastic helix, retrognatia, high arched palate, thin lips, dental abnormalities and large ears (Fig. 5). On her skin examination atrophic erythematous linear lesions and focal herniations of subcutaneous tissue were seen (Fig. 5). She also had other dysmorphic properties such as syndactyly of third and fourth fingers of the right hand, sacral dimple, pes planovalgus and overriding of toes (Fig. 5). Opthalmologic examination revealed optic atrophy, hypermetropia and astigmatism.

The metabolic work-up was unremarkable, including analysis of ammonium, lactate, acylcarnitine profile, amino acids, pyruvate, biotinidase activity. Creatine kinase level and thyroid functions were normal. Nerve conduction studies were normal. Her skeletal survey showed bipartite and hypoplastic right clavicula, bifid appearance of the fourth right rib, increased concavity of the first left rib and subluxation of the left proximal radius. Her abdominal ultrasonic showed smaller sizes of liver, spleen and kidneys according to age. She had normal karyotype. At last follow-up at 9.5 years old, she did not have head control and was unable to sit unsupported. She had been seizure-free for two years, her EEG showed multifocal epileptic abnormality and she was receiving oxcarbazepine and valproic acid for seizures and baclofen for spasticity.

### Molecular genetic studies

Patient 1 was whole exome sequenced (WES) as part of the BBMRI large prospective cohort (https://cordis.europa.eu/project). In brief, exome capture was performed using the Agilent SureSelect Human All Exome V5 kit, and sequencing performed on an Illumina HiSeq4000 at the Centro Nacional de Analisis Genómico, Barcelona. Patient 2 and family members were recruited at the Department of Paediatric Neurology, Izmir, Turkey. WES was performed by the Genomics Platform at the Broad Institute of MIT and Harvard, Cambridge, USA as part of the Centre for Mendelian Genomics. Libraries were created with an Illumina exome capture (38 Mb target) and sequenced with a mean target coverage of > 80×. Exome sequencing data for both patients were processed and analysed on the RD-Connect Genome-Phenome Analysis Platform (https://platform.rd-connect.eu/genomics). Likely pathogenic variants, affecting the function of the gene, and potentially causing disease, were identified applying standard filtering criteria: minor allele frequency < 1%, and high to moderate variant effect predictor (i.e. nonsense, splice site, frameshift, in-frame and non-synonymous variants). Shortlisted variants were interrogated for their predicted in silico deleteriousness and previous known association with human disease [49].

PORCN cDNA extracted from fibroblasts derived from patient 1 was amplified using the ImmolaseTM DNA polymerase Kit (Firma Bioline) and primers for exon 4 and exon 13 (Primer sequences are 4F: CAGTGGAGTTCATGGGCTACC and 13R: ATCGACATCAAACAGGGAGCC). Hereby, 40 ng of cDNA for each sample were used. PCR was following a standard protocol with 40 cycles of amplification. Sanger sequencing was performed using the BigDye Terminator v1.1 cycle sequencing kit (Applied Biosystems) on a 3100 genetic analyzer (Applied Biosystems).

### Skin biopsy and fibroblast culture

Fibroblasts were isolated from a fresh skin biopsy derived from patient 1 following standardized Eurobiobank protocols. In brief, biopsies were washed with sterile PBS and digested at 37 °C for 15 min with 2.5% trypsin (Thermo Fisher Scientific) and a further 90 min with 0.5% collagenase (Type IV, Sigma-Aldrich). Fibroblasts were then proliferated in Ham’s F-10-Complete Medium (Thermo Fisher Scientific) supplemented with 20% fetal bovine serum (FBS, SeraLab–BioreclamationIVT), 2% penicillin–streptomycin (Thermo Fisher Scientific), 1% GlutaMAX™ (Thermo Fisher Scientific) and 1% Fungizone (Thermo Fisher Scientific). Once fibroblast cells attained sufficient confluency, they were frozen and stored long-term in liquid nitrogen tanks at − 196 °C.

Fibroblast cell lines of patient 1 and controls were cultured in DMEM containing 10% fetal calf serum (both from Gibco). Gender- and age-matched control cell lines were obtained from the MRC biobank Newcastle [50]. When cells reached confluency of 70%, they were split using 1 × Gibco™TrypLE Express (Thermo Fisher Scientific) and re-grown to the required confluence. For proteomic analysis of the secretome (see below), cells were washed twice with PBS, serum-starved for 12 h, pulsed with 10% serum for 1 h followed by two further washing steps with PBS and replacement of the medium by DMEM without serum for 3 h. Next, medium was collected and snap frozen in liquid nitrogen and stored at − 80 °C until further use.

### Immunofluorescence studies

Fibroblasts derived from the *PORCN*-patient 1 as well as from sex and age matched controls were grown on 24-well glass coverslips until they reached a confluence of 70%. After culture medium was removed, cells were washed twice with PBS and fixed with ice-cold methanol for 10 min followed by a washing step with PBS. Next, primary antibodies were diluted 1:100 in PBS/0.1% Triton X100 (BiP: ab108615; Collagen 6 (VI-26): MAB3303; Collagen 6 (3C4): MAB1944; Lamin A/C: SC-7292; Periostin: ab 14,041; PORCN: ab105543; Vimentin: GTX112661), applied to fixed cells and incubated at 25 °C for one hour. Afterwards, cells were washed thrice with PBS/0.1% Triton X100 followed by incubation of secondary antibodies (Invitrogen: anti-rabbit-594, anti-mouse-594, anti-rabbit-488 and anti-mouse-488 each diluted 1:500 in PBS/0.1% Triton X100) for two hours at 25 °C. Next, samples were covered in fluorescence mounting medium (Dako).

### Immunoblot studies

Immunoblot studies on fibroblast whole protein extracts were performed as described previously [51]. For that purpose, following antibodies were used: α-ATF6 (Abcam, ab122897, 1:100), α-BiP/GRP78 (BD, #610978, 1:100), α-GRP94/Endoplasmin (Genetex, GTX103203, 1:100), α-GRP170/HYOU1 (Genetex, GTX102255, 1:100), α-LC-3 (Abcam, ab51520, 1:100), α-peIF2a (Genetex, GTX50300, 1:100), α-pIRE1 (Abcam, ab48187, 1:100), α-pPERK (Cell Signalling, #3179, 1:100), α-PORCN (Abcam, ab105543, 1:100), α-Ubiquitin (Abcam, ab19247, 1:100).

### MTT-assay

The MTT assay is a colorimetric assay for assessing cell metabolic activity, reflecting the number of viable cells present and was applied as described before [52] on control (n = 3; grouped) and *PORCN*-patient derived fibroblasts. For each cell line eight technical replicates were analyzed under basal conditions and after Tunicamycin-treatment (see above).

### Proteasome-assay

The proteasomal activity was analyzed making use of a commercially available kit (Abcam: ab107921) on control (n = 3; grouped) and *PORCN*-patient derived fibroblasts. For each cell line eight technical replicates were analyzed under basal conditions and after Tunicamycin-treatment (see above).

### Unbiased proteomic profiling of the secretome

#### Processing of pulse-chased cell culture medium samples

1 ml of the snap-frozen medium was thawed on ice, mixed with 3 ml of ice-cold acetone and incubated overnight at − 20 °C to precipitate proteins. After centrifugation at 4 °C for 20 min at 20.000 g, supernatant was discarded, and the precipitated proteins dried under a flow hood for several minutes. Next, the dried protein pellet was solubilized by adding 8 M urea and mixing for one hour. After complete dissolving, samples were diluted to 2 M urea utilizing 10 mM ABC (Ammonium bicarbonate, pH 7.8) buffer. To determine protein concentration, a BCA (Pierce BCA protein assay kit) was conducted according to the manufacturer’s instructions. Reduction and carbamidomethylation of the samples were carried out by using 10 mM TCEP (tris-(2-carboxyethyl)-phosphin) at an incubation time of 30 min at 37 °C and by using 15 mM IAA (iodacetamide) at an incubation time of 30 min at RT, respectively. Proteins were digested in solution using a 1:100 ratio of trypsin to protein. The tryptic-digest was conducted overnight at 37 °C and stopped the next day by adding 99% formic acid (FA). The samples were desalted using solid phase extraction with C18 filter cartridges (Waters), washed with 0.1% trifluoroacetic acid (TFA) and eluted with 80% acetonitrile (ACN). Cleaned samples were dried by using a vacuum concentrator. Concentration was adjusted to 1 µg/µl with 0.1% TFA.

All proteolytic digests were checked for complete digestion by using monolithic column separation (PepSwift monolithic PS-DVB PL-CAP200-PM, Dionex) on an inert Ultimate 3000 HPLC (Dionex) by direct injection of 1 μg sample. A binary gradient (solvent A: 0.1% TFA, solvent B: 0.08% TFA, 84% ACN) ranging from 5 to 12% B in 5 min and then from 12 to 50% B in 15 min at a flow rate of 2.2 μl/min and at 60 °C, was applied. UV traces were acquired at 214 nm [53].

#### Mass spectrometry data acquisition

The mass spectrometry measurements were conducted in data independent acquisition mode (DIA). For this purpose, each sample analyzed was mixed with an appropriate amount of iRT standard (Biognosys) peptides and 1 µg of each sample was subjected to the analysis. Samples were loaded on an Ultimate 3000 Rapid Separation Liquid chromatography (RSLC) nano system with a ProFlow flow control device coupled to a Fusion Lumos Tribrid mass spectrometer (both from Thermo Scientific). After initial loading, peptides were concentrated on a trapping column (Acclaim C18 PepMap100, 100 µm, 2 cm) using 0.1% TFA at a flow rate of 10 µl/min. Following sample separation was accomplished on a reversed phase column (Acclaim C18 PepMap100, 75 µm 50 cm) using a binary gradient: 3% solvent B (84% ACN with 0.1% TFA) for 10 min, a linear increase of solvent B to 35% for 120 min, a linear increase of solvent B to 95% for 10 min followed by a linear decrease of solvent B to 3% for 5 min. Full MS scans were acquired from 300 to 1100 m/z at a resolution of 60,000 (Orbitrap) using the polysiloxane ion at 445.12002 m/z as lock mass. The automatic gain control (AGC) was set to 5E5 and the maximum injection time to 20 ms. Full MS scans were followed by 30 DIA windows acquired at a resolution 30,000 (Orbitrap) with an AGC set to 1E6, a maximum injection time of 60 ms and a normalized collision energy of 32 (HCD).

#### Mass spectrometry data analysis

The acquired data were imported into the software Spectronaut (Biognosys). As proteome background the human proteome data was selected from UniProt (www.uniprot.org) containing 20,374 entries. The processing settings were set as following: enzyme was trypsin, the minimum and maximum peptide length was set to 7 and 52 respectively, missed cleavages were set to 2. Carbamidomethyl for cysteine was set as fixed modification and acetyl (protein N-term) and oxidation of methionine was set as variable modifications. All settings regarding the library generation including tolerances, identification, filters, iRT calibration and workflow were set to factory defaults. For the relative quantification, the option Top N max 3 was taken meaning that for each protein the average of the 3 most intense identified peptides are taken to give the protein the quantitative value. The files analyzed were searched against an in**-**house made spectral library [32] and protein identification was done by using the Pulsar search engine included in Spectronaut.

#### Visualization of regulated proteins and GO-terms

The voronoi diagrams depicting functional categories and proteins were created using the online software Proteomaps [54]. The size of a protein tile depends on the size of the calculated ratio. This was determined as the mean value of three biological replicates.

#### Protein network analysis

To study potential functional protein interaction in silico, the resulting list of regulated proteins was entered into the STRING database [55]. For this purpose, the significance value for the interactions in the STRING network was calculated by combining the categories co-expression, experimental, knowledge and text mining from the database.

## Supplementary Information

**Additional file 1.** Summary of clinical findings described in male *PORCN* patients.

## Data Availability

The spectral library data have been deposited to the ProteomeXchange Consortium via the PRIDE partner repository.
